# ‘RA and the microbiome: do host genetic factors provide the link?

**DOI:** 10.1016/j.jaut.2019.02.004

**Published:** 2019-05

**Authors:** Philippa M. Wells, Frances M.K. Williams, M.L. Matey-Hernandez, Cristina Menni, Claire J. Steves

**Affiliations:** a The Department of Twin Research and Genetic Epidemiology, King's College London, St Thomas' Hospital, Lambeth Palace Road, London, SE1 7EH, UK; b Clinical Age Research Unit, Kings College Hospital Foundation Trust, London, UK

**Keywords:** Genetics, Microbiome, Rheumatoid arthritis, Autoimmunity, Prevotella, Amplicon sequence variants

## Abstract

Rheumatoid arthritis (RA) is a chronic autoimmune disease, characterised by painful synovium inflammation, bony erosions, immune activation and the circulation of autoantibodies. Despite recent advances in therapeutics enabling disease suppression, there is a considerable demand for alternative therapeutic strategies as well as optimising those available at present. The relatively low concordance rate between monozygotic twins, 20–30% contrasts with heritability estimates of ∼65%, indicating a substantive role of other risk factors in RA pathogenesis. There is established evidence that RA has an infective component to its aetiology. More recently, differences in the commensal microbiota in RA compared to controls have been identified. Studies have shown that the gut, oral and lung microbiota is different in new onset treatment naïve, and established RA patients, compared to controls. Key taxonomic associations are an increase in abundance of *Porphyromonas gingivalis* and *Prevotella copri* in RA patients, compared to healthy controls. Host genetics may provide the link between disease and the microbiome. Genetic influence may be mediated by the host immune system; a differential response to RA associated taxa is suggested. The gut microbiome contains elements which are as much as 30% heritable. A better understanding of the influence of host genetics will shed light onto the role of the microbiome in RA. Here we review the role of the microbiome in RA through the lens of host genetics, and consider future research areas addressing microbiome study design and bioinformatics approaches.

## Highlights

• Rheumatoid arthritis (RA) affects 1% of the population and is highly debilitating.
• RA is ~65% heritable, yet the concordance rate between monozygotic twins is just 20–30%, indicating a substantive role of other risk factors.
• Studies have shown that the gut, oral and lung microbiome is different in treatment naïve and established RA patients, compared to controls.
• Current findings suggest an important influence of host genetics on the microbiome, which may contribute to RA via the host immune system.
• Associations of the microbiome with RA described thus far are confounded by host genetics, and future studies need to take account of this.

## Graphical abstract

Putative interactions mediating the link between host genetic factors, the gut and oral microbiome and rheumatoid arthritis.

**Figure:**
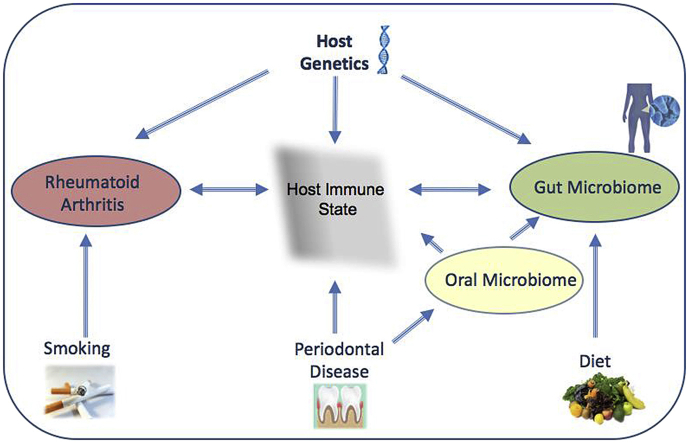

## 1 Introduction

Rheumatoid arthritis (RA) is a chronic multisystem autoimmune condition, characterised by painful swelling of the synovial joints bone and tendon damage. RA affects almost 1% of the population and is highly debilitating, with a profound effect on life quality in both young and elderly people. It has a significant economic impact on society partly through loss of working ability [1]. Despite considerable recent advances in therapeutics enabling disease suppression, there is still a sizeable minority of patients where drug therapies are ineffective or poorly tolerated, with potential serious adverse effects and the need for regular blood monitoring to early detect these [2]. Thus, there is a considerable demand for alternative therapeutic strategies as well as optimising those available at present.

RA is a common complex disease derived from the interplay between genetic and environmental factors [3,4]. Known risk factors include periodontal disease [5], smoking [4], diet [6] and hormone fluctuation – the disease is more prevalent in women [[5], [6], [7]]. The relatively low concordance rate between monozygotic twins, at 20–30%, contrasts with high heritability estimates of ∼65% [8], indicating a substantive role of other risk factors in RA pathogenesis in genetically susceptible individuals [9]. There is early evidence of epigenetic influence [10] and a longstanding appreciation of the possible role of infection as triggering the immune activity [11]. Despite genome-wide association studies (GWAS) and GWAS meta-analyses of increasingly large samples, the 349 variants identified for RA account for less than a third of the estimated heritability – a common finding in complex traits. This “missing heritability”, i.e. the inability to account for the proportion of phenotypic variance contributed by genetic factors – may be due to (i) structural variation (such as copy number variations-CNVs), (ii) by rare variants, or (iii) by environmental factors which are influenced by host genetics.

RA presents disease subsets, the clearest of which are characterised by the presence or absence of auto-antibodies – seropositive and seronegative RA respectively. There is a genetic aetiological difference, with seropositive RA being secondary to the human leukocyte antigen (HLA) DRB1 (encoding the major histocompatibility complex -MHC) – the shared epitope. Heritability differs by disease subtype, and is estimated at around 65 and 30% for seropositive and seronegative disease, respectively. Seropositive disease is much more clearly defined so will be the focus of this review. Symptoms develop in the later stages of immune dysfunction following a prolonged period of autoimmunity which, in seropositive RA, is marked by a surge in circulation of auto-antibodies – rheumatoid factor and anti-citrullinated protein [6]. Even in seronegative RA the onset of symptoms is typically preceded by years, or even decades, of increased levels of circulating pro-inflammatory cytokines [1]. This has prompted the theory that an environmental factor triggers disease progression. The microbiome, and particularly the alimentary tract microbiome, offers one such candidate mechanism [12].

The microbiome is the collective genome of the vast community of commensal micro-organisms - predominantly bacteria, which inhabit epithelial surfaces [5], blood [13] and tissue [14]. These organisms form a vast symbiotic community, the importance of which in physiological function has only recently been uncovered, secondary to availability of next generation sequencing [15]. The microbiome – the bulk of which is contained within the intestine [16], constitutes a substantial antigenic load and is essential for normal immune development in the neonate, with a life-long role in immune education [17]. It is therefore highly plausible that it has a role – possibly very early in life – in the promotion of subsequent autoimmunity. Further, the microbiome represents a unique physiological interface - a signalling hub, integrating environmental inputs with genetic and immune signals [15].

There is substantial diversity in the microbiome amongst the population [18], and individual differences may be influenced by diet [19], host genetics [20], age [21], host general health [22], antibiotic exposure and bowel habit. Specific variations associate with diseases, and to date the microbiome has been implicated in a vast array of medical conditions, including inflammatory bowel disease [23], cancer [24] and obesity [25]. The gut microbiome is the most influential, and are a number of ways that it can be altered therapeutically: pro or pre-biotics, diet and faecal microbiota transplantation, raising the possibility of hitherto unexplored treatment options.

Studies suggest that the microbiome and particularly the gut microbiome is different in people with RA and may be implicated in the pathogenesis of RA [18,26]. However, the many cross-sectional observations are challenging to place in the context of the temporal evolution of autoimmune disease which evolves over decades and is usually treated as soon as symptoms manifest and the diagnosis is confirmed. In RA there is a gradual transition to a pro-inflammatory phenotype, facilitating the development of disease in genetically susceptible individuals. There may be a bi-directional relationship between RA pathogenesis and the microbiome, in which the microbiome may contribute to the pro-inflammatory phenotype during the propagation stage of autoimmunity. To date nine epidemiological studies [5,18,26,35,44,[117], [118], [119], [120]] have shown alterations in the microbiome in both treatment naïve - excluding an influence of drug effects on the microbiome therefore, and established RA patients, with mechanisms demonstrated in mouse and cellular models. Taxa associations replicate across studies to a greater degree than is usually shown in disease specific microbiome studies, as shown by a recent meta-analysis [27].

Whilst host genetic factors clearly predispose to RA they may also mediate in part the interaction between the microbiome and RA pathogenesis. The gut microbiome itself contains elements which are as much as 30% heritable [20]. Given that the identified genetic risk loci in RA are associated with immune function, it is feasible that risk genotypes for RA act in part via the microbiome. Therefore, the microbiome may explain part of the aforementioned missing heritability in RA; microbes produce a range of enzymes, chemicals, hormones and vitamins that may interact with host metabolism, contributing to as much as one third of the metabolites identifiable in human blood [28].

In this review, the evidence for the role of the gut microbiome in RA will be evaluated, through the lens of host genetic factors. An overview of the genetic aetiology of RA will be given, followed by an evaluation of the evidence for the role of the microbiome in RA, and potential links between host genetics and the microbiome in RA. Areas for further research will then be considered.

### 1.1 Genetic aetiology of rheumatoid arthritis

**Fig. 1:**
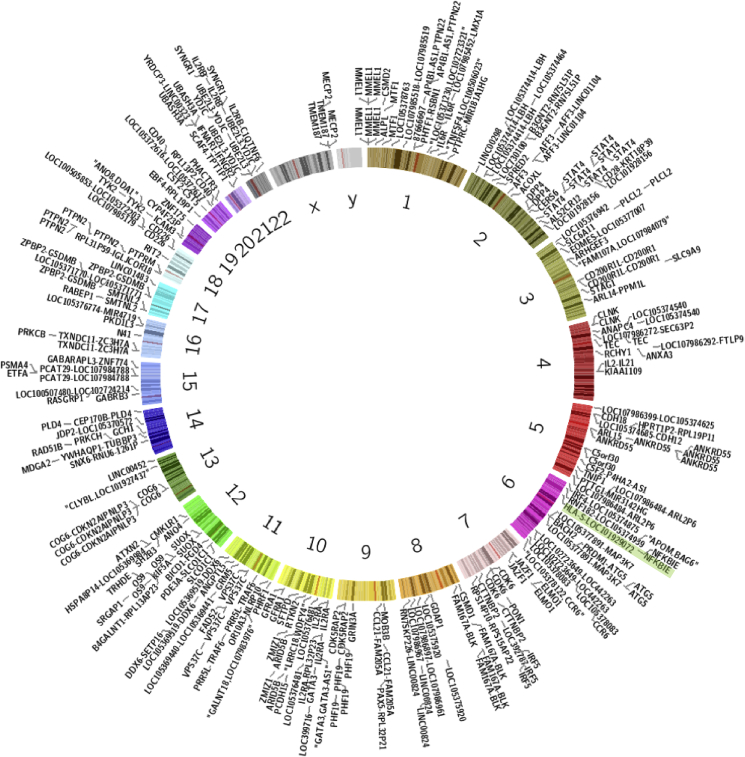
**Genetic Associations with rheumatoid arthritis shown to date.** HLA type (highlighted) shows direct mechanistic links with the oral and gut microbiome.

In the past few years large-scale human GWAS of RA having over n = 100,000 participants have led to the identification of a substantial number of associated genetic loci. Several common pathways associated with prevalence, severity and progression of rheumatologic disease have been identified. Forty GWAS found a total of 349 SNPs across all chromosomes (Fig. 1), that are associated with RA (Table 1), and 100 SNPs have been replicated on meta-analysis. These studies have revealed that the dominant risk loci in RA are, unsurprisingly for autoimmune disease, located in the MHC region [8] on 6p21.3 and these account for the major proportion of the current heritability explained in RA. Classically, this MHC association is explained mechanistically by the ‘shared epitope’ hypothesis [8]. The HLA-DRB1 shared epitope alleles - *04:01, *04:04, *04:05, *04:08, *10:01, *01:01 and *01:02 encode for amino acids in position 70 to 74 within the binding site of MHC-II; they therefore influence the host response to extracellular immune ligands. The shared epitope alleles are associated with increased susceptibility to RA, and particularly anti-citrullinated protein antibody (ACPA) positive RA; carriers of these alleles have enhanced immune response to citrullinated proteins. Heterozygosity for human leukocyte alleles (HLA) may confer greater risk of disease due to a wider auto-antigen repertoire of carriers. MHC variants have wider impact than the classic shared epitope hypothesis as this region is characterised by extremely dense and diverse sequence variations, and contains around 250 genes encoding numerous immune molecules in addition to HLA such as complement factors, cytokines and other proteins involved in antigen processing. The complexity of genomic control mechanisms across this region is enormous and only just being unravelled [8,29] (see Fig. 1).

**Table 1:** GWA studies of rheumatoid arthritis.

Study	Cohort				Genetic Association	Mapped Trait
	Ancestry	Cases		Controls		
WTCCC 2007 [81]	European		1860	2938	HLA-DRB1, PTPN22, MHC	RA
Plenge et al., 2007 [82]	European		1522	1850	TRAF1-C5, PTPN22, HLA-DRB1, IL6ST, ANKRD55, SPRED2, C5orf30 PXK, RBPJ, CCR6, IRF5, AFF3, CCL21, IL2RA, CD247, IL2, IL21, SH2B3, BATF, IKZF3, UBASH3A, TNFRSF14, PTPN22, REL, AFF3, STAT4, CTLA4, TNFAIP3, TRAF1, C5, PRKCQ, CD40, POU3F1, KIF3, HLA-DRB1	RA
Plenge et al., 2007 [83]	European		397	1211	TNFAIP3, OLIG3	RA
Liu et al., 2008 [84]	European		89	NR	MAFB, QKI, IFNK, LASS6, CST5, LMO4, CENTD1, PON1	response to TNF antagonist
Julia et al., 2008 [85]	European		400	410	SALL3	RA
Raychaudhuri êt al. 2008 [86]	European		3393	12,460	MMEL1, TNFRSF14, CDK6, CCL21, KIF5A, PIP4K2C, CD40, PRKCQ, PTPN22, HLA-DRB1, TNFIP3, OLIG3	RA
Cui et al., 2009 [87]	European		531	849		ACPA measurement
Gregerson et al., 2009 [88]	European		2418	4504	REL, CTLA4, BLK, PTPN22, TRAF1, C5	RA
Kochi et al., 2010 [89]	Japanese		2303	3380	CCR6, STAT4, TNFAIP3, OLIG3, HLA-DRB1	RA
Stahl et al., 2010 [90]	European		5539	20,169	IL6ST, ANKRD55, C5orf30, PXK, RBPJ, CCR6, IRF5, AFF3, CCL21, IL2RA, CD247, IL2, IL21, SH2B3, BATF, IKZF3, UBASH3A, TNFRSF14, PTPN22, REL,AFF3, STAT4, CTLA4, TNFAIP3, TRAF1, C5, PRKCQ, CD40, POU3F1, KIF3, HLA-DRB1	RA
Padyukov et al., 2010 [91]	European		1147	1853	HLA	RA
Freudenberg et al., 2011 [92]	Korean		801	757	BLK, TRHDE, ARHGEF3, HLA-DRB1, PADI4	RA
Terao et al., 2011 [93]	Japanese		1247	1486	AIRE, PFKL, HLA, PADI4	RA
Hu et al., 2011 [94]	Korean		100	600	APOM	RA
Okada et al., 2012 [95]	Japanese		4074	16,891	B3GNT2, ANXA3, CSF2, CD83, NFKBIE, ARID5B, PDE2A, ARAP1, PLD4, PTPN2, ETS1, FLI1, GCH1, PRKCH, ZNF774, PRKCB1, IRF8	RA
Wang et al., 2012 [96]	European		1157	NR	SPSB1, SLC6A11, ENOX1, MDGA2, ENSG00000102921, PSMA4, RCHY1, EFTA	RA response to drug
Krintel et al., 2012 [97]	European		196	NR	NR2F2, MAP2K6, ALPL, CBLN2, QPCT, CNTNAP4 PDE3A, SLCO1C1	RA, response to TNF antagonist
Myouzen et al., 2012 [98]	Japanese		2303	3380	NFKBIE, RTKN2	RA
Eyre et al., 2012 [99]	European		3297	15,870	HLA, ANKRD55, MMEL1, REL, SPRED2, AFF3, STAT4, CD28, CTLA4, RBPJ, GIN1, TNFAIP3, IRF5, CCL21, TRAF1, IL2RA, DDX6, CD40, PADI4, POU3F1, GATA3, ARID5B, CD5, VPS37C, RASGRP1, TLE3, IRF8, IKZF3, RCAN1, RUNX1, MMEL1, SPRED2, AFF3, TAGAP, IRF5, IRF8, IKZF3, GSDMB, ORMDL3, RCAN1, RUNX1, AFF3, CTLA4, ICOS, RBPJ, IKZF3, GSDMB, ORMDL3, IRAK1, TMEM187, HCFC1, STAT4, SLC9A9, CD28, CTLA4, PTPN22, RSBN1, IL2RB, TYK2, RAVER1, ICAM3, REL, DNASE1L3, PXK, GIN1, C5ORF30, BLK, CCR6, PTPN2, ANKRD55, BACH2, ELMO1, IL6R, AFF3, TRAF1, CTLA4, ICOS, IKZF3, GSDMB, ORMDL3, IRAK1, TMEM187, HCFC1, IL2RB, TYK2, ICAM3, RAVER1, DNASE1L3, PXK, GATA3, GIN1, C5orf30, DDX6, SETP16, TNFSF18.TNFSF4, TAGAP, SPRED2, TNIP1, ANKRD55, CD2, COG6, IL6R, ACTN1	RA
Cui et al., 2013 [100]	European		2706	NR	4 SNPs, genes not reported.	RA response to TNF antagonist
Negi et al., 2013 [101]	North Indian		706	761	ARL15, HLA-DQA2, HLA-DQB1, C6orf10, HLA-DQA1	RA
Okada et al., 2013 [29]	European East Asian		14,361 4873	42,923 17,642	ACOXL, AFF3, ANKRD55, ARID5B, ATG5, ATM, BLK, C1QBP, C4orf52, C5orf30, CL19, CCL21, CCR6, CD2, CD226, CD28, CD40, CD5, CD83, CDK2, CDK4, CDK6, CEP57, CASP8, CFLAR, CLNK, COG6, CTLA4, CXCR5, ABHD6, PXK, DNASE1L3, EOMES, ETS1, FADS1, FADS2, FADS3, FCGR2A, FCRL3, GATA3, GRHL2,HLA-DRB1, IFNGR2, CSF3, IKZF3, IL2, IL21, IL20RB, IL2RA, IL2RB, CSF2, IL3, IL6R, IRAK1, IRF4, IRF5, IRF8, JAZF1, LBH, LOC100506023, LOC145837, LOC339442, MED1, MTF1, INPP5B, NFKBIE, P2RY10, PADI4, PLCL2, AHNAK2, PLD4, PPIL4, PRKCH, PRKCH, PRKCQ, PTPN2, PTPN22, PVT1, RAD51B, RASGRP1, RCAN1, REL, RTKN2, RUNX1, LOC100506403,SFTPD, SH2B3, PTPN11, SPRED2, STAT4, SYNGR1, TAGAP, TEC, TNFAIP3, MMEL1, TNFRSF14, TNFRSF9, TPD52,TRAF1, C5, TRAF6, RAG1, RAG2, TXNDC11, TYK2, YDJC, UBE2L3, WDFY4, ZNF438, PADI4, B3GNT2, CLNK, CSF2, IL3, HLA-DRB1, NFKBIE, AHNAK2, PLD4, IRF8, UBASH3A, P2RY10, LOC339442, FCGR2A, CASP8, CFLAR, ANKRD55, PVT1	RA
Orozco et al., 2014 [102]	European		3034	5271	TNFRSF14	RA
Bossini-Castillo et al., 2014 [103]	European		1148	6008	GRM5, RNASEH2B, FAM124A, CLYBL, MICA, HLA-B, HLA-DRB1, HLA-DQA1, SMIM21	RA
Kim et al., 2014 [104]	Korean European		2234 10,288	7065 35,502	UBASH3A, ETS1, FLI1, TNFSF4, SYNGR1, LBH, COG6, RAD51B, EOMES, MMEL1, PADI4, POU3F1, PTPN22, CD2, IL6R, FCRL3, FCGR2A, PTPRC, REL, SPRED2, AFF3, STAT4, CD28, CTLA4, RPP14, C4orf52, RBPJ, ANKRD55, C5orf30, OLIG3, TNFAIP3, TAGAP, CCR6, IRF5, BLK, CCL21, TRAF1, IR2RA, PRKCQ, GATA3, ARID5B, CD5, DDX6, OS9, RASGRP1, KIF23, TLE3, IRF8, IKZF3, GSDMB, PTPN2, TYK2, CD40, RCAN1, RUNX1, UBE2L3, IL2RB, TNFAIP3	RA
Senapati et al., 2014 [105]	Indian		281	157	PPM1L, ARL14, PTPRM	response to methotrexate, RA
Jiang et al., 2014 [106]	Han Chinese		952	943	SPRED2, AFF3, CCR6, TRAF1, DPP4, CDK5RAP2, DPP4, MHC HLA-DQA2, HLA-DQB1	RA
Govind et al., 2014 [107]	South African		263	374	LOC100131866, NR5A2, KIAA1542, HLA-DRB1	RA
de Rooy et al., 2015 [108]	Western European		262	NR	HUNK, SCAF4, AP000255.6–001	RA, ACPA, joint damage measurement
Honne et al., 2016 [109]	Japanese		282 moderate 131 severe	31 mild RA	MAP3K7, BACH2, WDR27, GFRA1, CSMD2, SMARCAL1 NLRP10, PKD1L3, IST1, ATXN1L, SMARCAL1, CDH18	RA response to TNF antagonist
Julia et al., 2016 [110]	Spanish		896	282	IRX1	RA, RF
Marquez et al., 2016 [111]	European		3911	10,398	COG6, PTTG1, PTPN22, ATG5, TNFAIP3, TNPO3, IRF5, BLK, ICAM3, TYK2, UBE2L3	RA, SLE
Saxena et al., 2017 [112]	Arab		283	221	HLA-DRB1, CDH6, SMTNL2, GGT6, CD200R1	RA
Bluett et al., 2017 [113]	European		62	175	GJA5, ACP6	response to methotrexate, RA,
*Joo* et al., 2017 [114]	Korean		385	326	SLA, TG, SRGAP1	joint damage measurement
Wei et al., 2017 [115]	European		3323	15,785	ANKRD55, HLA-DQB1	RA
Yoo et al., 2017 [116]	Korean		120	118	TGFA	RA disease progression

Over 100 associated non-MHC loci have been identified through GWAS meta-analyses [29]. These include variants at the gene encoding protein tyrosine phosphatase, non-receptor type 22 (PTPN22) which functions as a negative regulator of T cell receptors [30], peptidylarginine deiminase 4 (PADI4) which encodes enzymes active in protein citrullination [31], signal transducer and activator of transcription 4 (STAT4) which encodes a transcription factor specific for T cell maturation [32], and TNFAIP3 which encodes tumour necrosis factor alpha (TNFα) [33]. These protein coding genes together influence the immune response and collectively promote a shift to a pro-inflammatory phenotype and increased sensitivity to immune stimulation [34]. Walsh and colleagues characterised the role of protein coding RA associated SNPs and showed involvement in both the innate and adaptive immune systems, which would support a shift to a pro-inflammatory phenotype: jak-STAT signalling pathway; IL-12 mediated signalling; endocytosis; T cell signal transduction; signalling downstream of interleukins and T cell receptors; cytokine signalling; cell adhesion molecules and B lymphocyte cell surface molecules. In keeping with most common complex trait GWAS results, the associated SNPs largely do not reside in protein coding regions, and may act distally with unidentified genes. Non-coding variants are likely to have a role in regulation of immune mediating gene expression [34].

However, as with other complex traits, a full understanding of genetic risk in RA has proved evasive; known risk loci explain only 15% of the estimated heritability, indicating that numerous associations are yet to be discovered [8]. This is perhaps because GWA studies were designed to detect common genetic loci associated with disease, yet genetic risk in RA may be mostly driven by rare variants, with a minor allele frequency (MAF) of less than 0.05 giving accumulated aetiological effect. Research methods must accommodate the polygenic nature of RA, with variable genetic architecture between individuals who may possess numerous RA associated variants of modest effect. Polygenic risk scores (PRS) provide a weighted genetic risk score for individuals, combining individual genotype data with the strength of the disease association for each risk variant. Association of PRS with phenotypes can be modelled, offering an effective option for RA research, and provides a surrogate model of RA allowing examination of the host genetic factors without the confounding influence of the disease or its treatment.

### 1.2 Gut microbiome in RA

Studies have shown that the gut [18], oral [5], and to a lesser extent, lung [35] microbiomes have been implicated in RA when comparing RA patients to healthy controls (see Table 2). Whether this association is causal has not yet been established. The gut microbiome has been the focus of the RA microbiome link - it constitutes over 80% of the total microbial biomass, with the closest links to the immune system. Nine studies have reported changes in diversity and taxa present in the microbiome of RA patients compared to age, gender and weight matched controls. Whilst lower gut microbiome diversity is known to be a generalised feature of disease [22], the taxonomic and bacterial gene associations with RA are of greater etiological interest. However, there are discrepancies in these associations across the published studies. A link with host genotype, mediated by HLA type in addition to a more general pro-inflammatory genetic predisposition in RA, is suggested.

**Table 2:** Studies of the microbiome in RA patients.

Study	Site	Cases	Controls	Methods
Shinenbaum et al., 1987 [117]	Gut	25 RA	25 healthy	Bacterial culture
Eerola et al., 1994 [118]	Gut	74 RA	91 healthy	Gas liquid chromatography analysis of bacterial cell fatty acid.
Toivanen et al., 2002 [44]	Gut	25 early RA	23 neuroinflammatory pain	16SrRNA oligonucleotide probes
Vaahtuvuo et al., 2008 [119]	Gut	50 RA	50 Fibromyalgia	Flow cytometry, 16s rRNA hybridisation, DNA staining
Scher et al., 2012 [5]	Oral	31 early RA 34 treated RA	18 healthy	16s
Scher et al., 2013 [26]	Gut	44 early RA 26 treated RA 16 psoriatic arthritis	28 healthy	16s
Liu et al., 2013 [120]	Gut	15 RA	15 controls	Quantitative RT-PCR
Zhang et al., 2015 [18]	Gut/Oral	94 early RA 21 treated RA	97 healthy	Metagenomes
Scher et al., 2016 [35]	Lung	20 RA 20 Sarcoidosis	28 Healthy	16s

Prevotella *copri* (P.*copri)* is the most frequently reported bacterial species showing variation of abundance between RA patients and unaffected controls. However, P*.copri* is associated with other inflammatory conditions including metabolic syndrome, insulin resistance, type II diabetes and atherosclerosis [36], in addition to RA [37,38], and may thrive relative to other bacteria within an inflammatory host environment [39]. Therefore, individuals with RA risk genotypes, leading to pro-inflammatory immune phenotype, may potentially constitute an ecological niche. This could be in addition to a possible role in disease causation, but needs to be borne in mind when assessing association studies*.*

Scher and collaborators performed 16s analysis on faecal samples from 144 participants – new onset RA (n = 44), chronic RA (n = 26), psoriatic arthritis (n = 16) and healthy controls (n = 28). They found that in the gut microbiome *P.copri* was most abundant in patients with new-onset RA compared with those with chronic treated RA (p < 0.01), psoriatic arthritis (p < 0.01) or healthy controls (p < 0.01) [26]. This important finding was partially replicated in the study below by Zhang and co-workers [18]. Higher abundance of *P. copri* in the gut microbiome is a characteristic on new-onset RA, they suggest, in which inflammation is relatively unabated by medication. Increased abundance of *P.copri* in new onset RA patients correlated with a decrease of *Bacteroides fragilis* (*B. fragilis*)*,* an important regulator of regulatory T cell (Treg) function. Tregs function in the establishment and maintenance of immune tolerance. This therefore suggested that *P.copri* may influence RA pathogenesis via indirect suppression of Tregs via the lower relative abundance of *B. fragilis* in these patients, but might equally be explained by the inflammatory milieu hypothesis. Genotyping of new onset RA patients showed that this increase was associated with HLA DRB1 4 shared epitope (SE) genotype [26]. This work replicated previous findings which - whilst not directly linked to RA, have linked host genetics with *Prevotella* and other taxa - *Pasteurellaceae*, and *Leptotrichia*, which were associated with SNPs encoding for ATP-binding cassettes, protein synthesis, cell division, and tumour suppression [40]. Similar association of *Prevotella* with HLA has been shown in mice [41]. These findings warrant further investigation. We know that the host genome impacts the microbiome: if microbiome changes are also mediated by genotype we could speculate that microbiome alteration appears before clinical disease manifests, and perhaps lies on the causal pathway to RA.

Zhang and collaborators undertook shotgun metagenomic sequencing on 212 faecal samples from 3 groups: 77 untreated RA patients matched with 80 unrelated healthy controls, 17 untreated RA patients matched with 17 healthy related controls, and a third group of 21 DMARD-treated RA patients [18]. To delineate features of the RA-associated gut microbiome, they identified 117,219 gene markers that were differentially enriched in RA patients versus controls and clustered the genes into metagenomic linkage groups (MLGs) on the basis of their correlated abundance variation among samples. They identified 88 MLGs that contained at least 100 genes, which separated RA-enriched and control-enriched MLGs. Of the MLGs comprising greater than 100 genes, RA was associated with MLGs containing *Lactobacillus salivaris, Clostridium asparagiforme, Gordonibacter pamelaeae, Eggerthella lenta, Lachnospiraceae bacterium, Bifidobacterium dentium, Lactobacillus spp* and *Ruminococcus lactaris*. The control group showed a negative correlation with *Haemophilus spp., K. pneumoniae* and *Bacteroides spp*., *B.bifidum* and *R.lactaris* [18], suggesting an antagonistic or mutually exclusive relationship and highlighting the interdependency of species especially when measured as relative abundances [42,43]**.** None of the MLGs containing 100 or more genes contained *Prevotella*, however when the authors compared the MLGs with the NCBI *Prevotella* reference genome, there was a trend towards increased abundance of *P.copri* as a function of RA duration in the first year of disease onset [18]. *Prevotella* may therefore be a particularly variable taxon, where bacterial genes and associated functions vary to a greater extent than in other taxa.

Zhang and collaborators also showed that treatment with RA therapeutics – disease modifying antirheumatic drugs (DMARDs), in which the inflammatory response is abated, was associated with a “normalised” more diverse microbiome; this is in contrast to the general observation that medication intake associates with reduced diversity [22]. This effect was observed for Methotrexate, as the most widely prescribed DMARD. Other drugs used by the cohort – Leflunomide, Prednisolone, Hydroxychloroquine and Etanercept were not prescribed to enough participants to power analysis. Further study of specific DMARDS and at multiple time-points is needed. However, this supports the hypothesis that microbiome changes in RA are driven at least in part by systemic inflammation.

The above studies accounted for body mass index (BMI) and sex, which have known associations with gut microbiota and these factors showed no association with microbiota profile. The reported associations thus far are therefore unlikely to be driven by diet or obesity. However an influence of diet on RA via the microbiome is possible, and warrants further study. Similarly, whilst gender is controlled for, the independent role of gender in the link between the microbiome and RA has not been investigated. However an influence is feasible, via genetic differences (some associated SNPs lie on the sex chromosomes), diet and hormone fluctuation.

*Prevotella copri* is therefore the key candidate taxon in RA, with a finding of increased abundance in RA patients compared to controls replicated across 4 of 7 published epidemiological studies of the microbiome using next generation sequencing [5,18,26,44]. Moreover, *P.copri* was shown to mediate immune activation with 24% of RA patients having IgA or IgG responses to Pc-p27, an HLA-DR presented *P.copri* peptide, compared to 2% in healthy controls [37]. Further investigating the specificity of *P.copri* in immune activation in RA, *P.gingivalis* (shown previously to increase in RA), *Bacteroides fragilis* (shown previously to decrease in RA) and Escherechia coli (not previously implicated in RA), were shown to have no immune memory. While suggestive, the associations in human RA patients to date has been unable to elucidate whether the *Prevotella*-RA association is driven by host inflammation, or is in some part causal.

Mouse studies demonstrated that the *Prevotellecae* enriched microbiome of RA patients, when transferred to arthritis susceptible SKG mice, increases sensitivity to arthritis via activation of autoreactive T cells in the intestine. T cell differentiation and an increase in autoreactive T cells is a known driver of RA pathogenesis. Faeces from 3 RA patients and 3 healthy controls were analysed for microbiome composition, before transfer to SKG mice. After 20 weeks of colonisation, the total number of CD4^+^ T cells and the number of pro-inflammatory IL-17 producing CD4^+^ T cells in the large intestine were increased in RA-SKG mice compared with HC-SKG mice [45]. It remains unclear whether *P. Copri* is robustly associated with or actually causative in RA [16,26,36,37,39,46]. One explanation for the conflicting findings is that *Prevotella* is a particularly variable taxon, with different genes and biological characteristics even at the strain level, leading to different results in relation to *P.copri* in RA. The jury is therefore still out on the relevance of prevotella to RA pathogenesis, though current findings are promising. Associations with other taxa show weaker evidence, with lower reproducibility across studies. Overall, taxa associations, whilst useful, can only go some way to indicating the genomic functional capacity of the microbiome. Further work, especially incorporating metagenomics analysis of genetic capability of a microbiome will be critical to provide clarity.

#### 1.2.1 CARD 9

The *caspase recruitment domain family member 9* gene (CARD9) which encodes an adaptor protein that integrates signals downstream of pattern recognition receptors [50], has been shown to be important in mouse models of arthritis [51]. Using two CARD9 knockout mouse models, with neutrophil-specific deletion, and wild-type control mice, the effect of CARD9 deficiency was investigated using the KBXN serum transfer model. Clinical signs of arthritis were quantified in CARD9 knockout mice which showed significant ankle thickening (P = 0.0047) and reduced grip strength (P = 2 × 10^−4^), compared to wild-type mice [51]. To date, no GWAS has shown CARD9 to be a risk allele for RA in humans, but it is an interesting model gene which has relevance in other autoimmune diseases including inflammatory bowel disease (IBD) [52], ankylosing spondylitis [53], IgA nephropathy [50] and colitis [54].

CARD9 knockout mice exhibited downregulation in IL-22 signalling, resulting in impaired recovery from colitis when compared to wild-type mice. Analysis of the gut microbiota revealed no difference in beta diversity, but highlighted a decrease in abundance of *Adlercreutzia* (genus), *Actinobacteria* (phylum), and *Lactobacillus reuteri* (species), indicating an in influence of CARD9 genotype on the gut microbiome in these mice*.* Transfer of the CARD9 knockout mouse microbiota to germ-free wild type mice resulted in an exacerbation of colitis, to a similar degree as CARD9 knock out mice – suggesting a causative, rather than correlative, relationship between the microbiome and IL-22 mediated inflammation.

The gut microbiota of CARD9 knockout mice showed impaired catabolism of tryptophan, a downstream effect of which is IL-22 production, and this is suggested to be a key underlying mechanism [54]. IL-22 is a Th17 cytokine, which in addition to an integral role in maintaining the gut barrier, has diverse functions in balance with pro-inflammatory IL-17, which vary according to tissue type and duration of expression. IL-22 promotes wound healing and tissue homeostasis acutely, however chronic unabated expression is associated with a number of inflammatory conditions. The role of neutrophils in RA has been highlighted in a recent review [55].

### 1.3 The oral microbiome in RA

#### 1.3.1 Shared epitope interaction with the oral microbiome

One of the earliest links between commensal microbiota and RA pathogenesis was shown in the oral microbiome. Individuals with RA had a higher incidence of periodontal disease – linked to oral microbiome dysbiosis, and that periodontal disease treatment improved RA symptoms [5,47,48]. This is highly plausible because of the known interaction between the oral microbiome, co-occurring with periodontal disease and progression to clinical RA, secondary to shared epitope HLA-DRB1 genotype. The oral and lung mucosa have been proposed to be the primary sites of protein citrullination in RA, via oral microbiome changes [5] and smoking status [4], respectively. The host immune response to citrullinated proteins is mediated by the shared epitope, which encodes the binding motif of MHC II. Variation in MHCII results in altered immune response to extracellular antigens, and carriers of the shared epitope have enhanced response to citrullinated proteins, and subsequent increase in ACPA. RA patients have been shown to have a 5.7 fold increased risk of periodontal disease (95% CI 2.35–13.84) in a stepwise logistic regression, including other predictors of periodontal disease – age, education, smoking, alcohol consumption level and BMI, only age and RA remained as significant predictors [48]. The oral microbiome may therefore be the primary mediator of protein citrullination, having greater influence even than smoking.

**Fig. 2:**
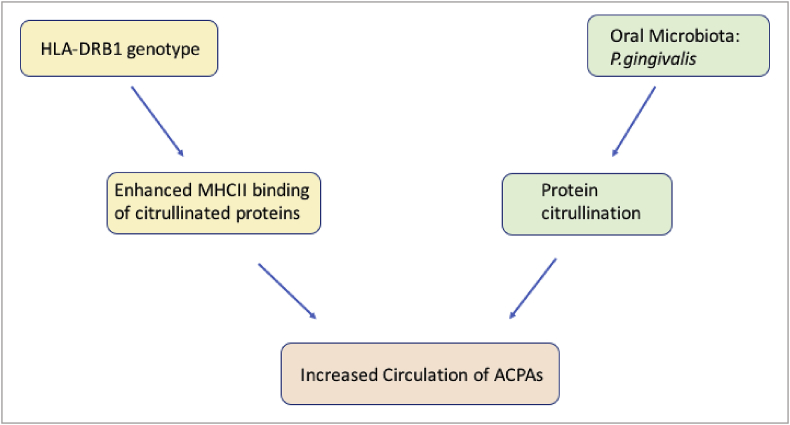
**Interaction between host genetics and the oral microbiome in RA.** Mechanism by which HLA-DRB1 genotype and *Porphyromonas gingivalis* in the oral microbiota can interact to contribute to RA pathology, via upregulation of circulating ACPA.

Bacterial taxa associated with RA may provide the mechanistic link for this association. *Porphyromonas gingivalis, is* an oral commensal found in increased in abundance in RA patients [5,18], and it is active in citrullination of host proteins – providing a precursor step for production of specific antibodies [18], and mediating synovial inflammation (see Fig. 2) [46]. Further, *Porphyromonas gingivalis* has evolved to alter its microenvironment within the oral microbiome by modulating the host TLR2 pathway to uncouple bacterial clearance from inflammation and therefore exacerbate the microbial ecological niche [49].

### 1.5 General mechanisms linking the microbiome and RA

**Fig. 3:**
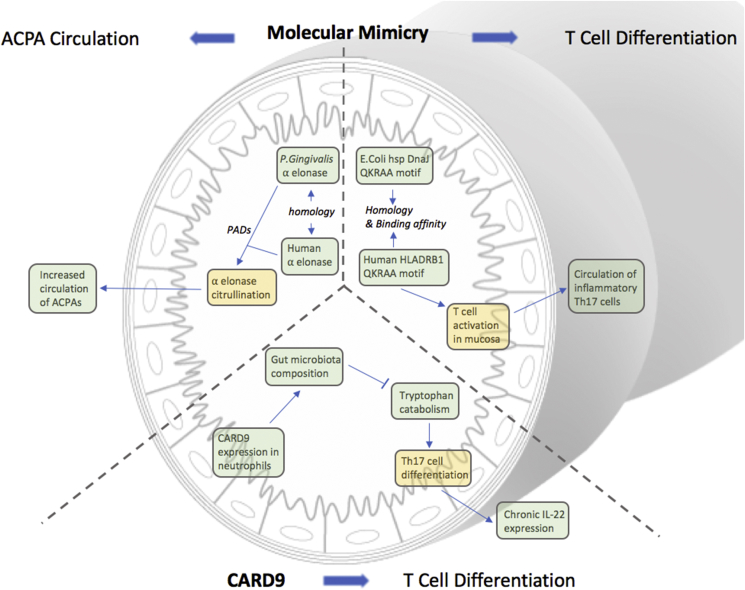
Summary of mechanisms by which molecular mimicry of the gut microbiota, and CARD9 genotype can contribute to systemic inflammation.

The accumulating evidence implicating the microbiome in RA pathogenesis has prompted investigation into the underlying mechanisms, of which a number have been proposed: molecular mimicry; outer membrane vesicles (OMVs); T cell differentiation; epigenetic modification; immune priming (see Fig. 3), and a role in immune ageing (see Box 1).

Box 1 **RA as a Model of Ageing – Potential role of epigenetics and the microbiome**

Patients with RA prematurely show a number of features of an aged immune system, and RA has therefore been proposed as a model of immune ageing. There are a wide range of disruptions to the delicate balance within the immune system which occur with ageing, mediated in part by epigenetic changes, and having potential impact on the host response to the microbiome [56]. Key changes are compromising of epithelial layer integrity through disruption of tight junctions [57], and immune-senescence [56,58], which may be secondary to a reduction in telomerase [56,59]. Loss of function of telomerase in lymphocytes, leads to loss of the unique ability of these cells to elongate telomeres, a consequence of which is cellular senescence [60]. A second pathway, independent of telomerase, is senescence secondary to genomic instability and prolonged activation of the DNA damage response (DDR). The DDR pathway has been shown to be particularly important in peripheral blood mononuclear cells (PBMC) and both naïve and memory CD4^+^ T cells, but not neutrophils, in RA patients [61]. Cellular senescence of T cells results in clonal expansion of sets of naïve T cells. Early in the onset of RA there is clonal expansion of both CD4^+^ T and C8+ T cells. This is accompanied by a relative lack of expression of co-stimulatory molecules from memory T cells, particularly CD28, which is required for efficient T-cell activation and proliferation. A lower level of CD28 is associated with a pro-inflammatory phenotype, increased cytotoxicity and increased rates of tissue infiltration [56,61]. Other immune changes in both ageing and RA include altered patterns of DNA methylation and therefore gene expression, chromatin remodelling, failure of protein homeostasis, altered nutrient sensing and mitochondrial dysfunction [56]. There are therefore diverse differences, having far reaching physiological effects. Animal models have been used to determine which intracellular pathways are most implicated in ageing, and a key identified difference is defective transgenic growth factor β (TGFβ) signalling. More specifically, downstream, SMAD3 signalling has been shown to promote cartilage damage [62]. The microbiome may contribute to these shifts in immune phenotype; expansion of the pro-inflammatory cytokine milieu in the host is suggested as the primary mechanism of immune ageing [63].

Alt-text: Box 1

The gut microbiome produces a variety of metabolites, including small organic acids, bile acids, vitamins, choline metabolites, and lipids [64,65]. The plethora of small molecules produced, alongside microbial cellular components, share some structural similarity with the host. Such molecular mimicry refers to the similarity of bacterial peptides of RA associated antigens, or to affinity of bacterial peptides to host receptors [18]. Molecules associated with bacterial cell to cell communication – quorum sensing, may also influence cellular processes within the host (see Box 1).

For example, *P.gingivalis,* which is found in increased abundance in the oral microbiome in RA (and also found in the gut) shares 82% homology of α-enolase with human α-enolase at the immunodominant region. Human antibodies against bacterial elonase also recognise human α-enolase, promoting further antibody production [66]. In RA patients, levels of anti-citrullinated human α-enolase antibodies and bacterial α-enolase are shown to correlate with one another (R^2^ = 0.0803, P < 0.0001) [67].

Molecular mimicry also promotes autoreactive T cell activation and proliferation. *E. coli* heat shock protein DnaJ contains a QKRAA motif that is also present in the HLA-DRB1 shared epitopes [66]. DnaJ strongly activated RA synovial T cells which had passed the positive selection in the thymus through weak binding with the corresponding HLA epitopes [68].

OMVs produced by gram negative bacteria modify the local environment to facilitate bacterial proliferation, signal between bacterial species and have been shown to communicate directly with host cells [[69], [70], [71]]. In the host, OMVs affect intracellular signalling [71] and overall metabolic profile [72]. Thus, investigation of OMVs may be an important step in understanding the link between the microbiome and host. Interestingly, OMVs of pathogenic and non-pathogenic strains of the same species manifest differing metabolic associations [73].

The anti-inflammatory influence of the microbiome may also play a role in RA. Dietary poly- and oligosaccharides resistant to upper gut digestion pass to the distal gut where they serve as a source of carbon and energy for gut bacteria. Through fermentative reactions, the gut microbiota can metabolize complex carbohydrates to produce small organic acids, the majority of which are comprised of the short-chain fatty acids (SCFAs) -acetate, propionate, and butyrate.

SCFAs, and butyrate in particular may influence host physiology, as these metabolites are linked to expansion of Tregs, and a protective anti-inflammatory role is proposed for them.

### 1.5 Considerations for future studies

#### 1.5.1 Limitations of mouse models in microbiome research

Mouse models are widely used in microbiome research and can be informative – particularly when used to understand or replicate a specific mechanism. However, there are key differences between mouse and human microbiome physiology which are seldom discussed. The mouse and human genome are separated by more than 90 million years of evolution, during which there has been substantial change in the immune system and its regulation. The GI tract anatomy and physiology of the mouse is markedly different to that of humans, and in particular the presence of the glandular forestomach in the mouse, with its biofilm of *lactobacillus spp,* and mucus trap where mucus and bacteria are recycled to the cecum. There are differences in morphology and retention time, and mice engage in coprophagy, and transfer gut microbiota between each other when housed together. These factors confer differences in mouse vs human microbiome, and in physiological response to bacteria. The presence of taxonomic differences in the murine versus human microbiome may have resulted in over-interpretation of the clinical relevance of findings shown in mouse models. There are numerous references to the importance of *segmented filamentous bacteria* (SFB) within the RA microbiome literature, since it was shown that introduction of SFB in a mouse model regulated TH17 differentiation [74]. This is often cited as convincing evidence of the microbiome inducing T cell differentiation. However, SFB, also known as *Candidatus arthromitus* within the Greengenes database, are usually only present in humans during early life, and so the relevance in human RA is at best unclear, and whether other species have comparable effect in humans remains open to debate.

In murine models of inflammatory arthritis germ free mice fail to develop the diseased state [45], in contrast to microbiota exposed arthritis model mice which ubiquitously develop symptoms, suggesting an integral role in immune development of commensal microbiota [75]. Germ free mice exhibit immune differences, culminating in a much-dampened immune response. Therefore, it is unsurprising that these mice do not develop inflammatory or autoimmune disease. The relative disease progression of mice gavaged with RA-associated microbiota compared to germ free mice should therefore be interpreted with caution and comparison to mice with a different (“healthy”) humanised microbiome might be preferable (see section 1.5.2).

Large-scale human observational studies in RA are underway, including the use of family- and twin studies to unpick the contributions of host genetics and microbiome. In addition, population based studies linked to health records are now incorporating microbiome assessment, which will allow the interrogation of the microbiome prior to diagnosis retrospectively. Studies in at risk groups, such as those at genetic risk, those with periodontal disease and smokers may provide insight as to the temporal relationship between microbiome alterations and altered inflammation. Low-risk interventional studies in humans are around the corner and will be needed to ascertain whether effects are relevant to the development of RA or bystanders of an altered milieu.

#### 1.5.2 Developing bioinformatic approaches for microbiome data

Next generation sequencing has provided the means to access a wealth of information relating to commensal bacterial communities, however there are a wide variety of technological and analytical methods available – it is important to understand the benefits and limitations of the most frequently used methods: operational taxonomic units (OTUs), amplicon sequence variants (ASVs) and metagenomics. The most frequently used approach in microbiome sequencing is16s. This refers to sequencing of bacterial ribosomal RNA (rRNA) gene, using methods which take advantage of the particular structure of the 16s ribosomal component [76]. 16s rRNA is a component of 30s small subunit of prokaryotic ribosome, of which there are 9 variable regions – varying between phylogenies, which are each flanked by highly conserved regions. DNA is first extracted from the biological sample. Following this, primers linked to an identifier barcode, specific to highly conserved binding sites, provide a template for PCR amplification. An alternative approach is whole genome metagenomic sequencing, this is more costly but provides important additional information - particularly bacterial genes present [77]. Classically, following sequencing data are assigned to operational taxonomic units (OTUs), in which sequences are binned (grouped) together according to a similarity threshold. There are a number of alternative methods picking OTUs – open reference, closed reference or de novo approaches can be taken. These approaches differ in the use of a database (e.g Greengenes or SILVA) as a reference when performing clustering. Within these approaches, a number of algorithms may be used. The de-novo approach, whilst computationally less efficient (requiring pair-wise comparisons between sequence reads), has been shown to produce OTUs that are more representative of functional microbial units [78]. The OTUs in conjunction with a genome reference database are then used to assign taxonomy, determining which bacteria are present in the samples. Amplicon Sequence Variants (ASVs), also known as exact sequence variants, offer an alternative approach to OTUs, and hold a number of advantages over traditional OTU methods [79]. Briefly, ASVs are generated by using the error rate within the dataset to infer true biological sequences, and group identical sequences exactly, rather than to a similarity threshold. There are a number of advantages to this approach, demonstrated by increased sensitivity and specificity of ASVs compared to the most often used methods of OTU generation [79,80]. ASVs overcome other key limitations of OTUs and allow for valid direct comparability between datasets [80], and accurate taxonomic assignment at the species level [79]. The direct comparability between datasets, in conjunction with correcting for ‘batch effects’ on analysis could allow for the merging of multiple RA microbiome datasets, which could shed light on the conflicting taxa associations reported. 16s analysis is inferior however to metagenomic sequencing, which although at greater expense, provides key additional information – genes present and full species assignment, allowing for much richer functional analysis. Moving forward, ASVs show exciting potential in 16s analysis, and could advance understanding of the role of the microbiome in RA, both through analysis of existing datasets and through use in future studies.

### 1.6 Sample collection, storage and processing

In most studies, the means of measurement of the gut microbiome is the microbial composition of stool samples. Although the faecal microbiome provides a useful indicator of the gut microbiome, they are of course distinct entities with differences starting in the mucosal layer, epithelium, lumen in small intestine through to stool. In studies of the gut microbiome therefore, a useful additional measure may be the use of colonic biopsy. Differences in sample storage, and particularly the immediacy of freezing sample, temperature on freezing or use of an RNA inhibitor with sample add to the difficulty of comparing results across studies. This is difficult to address, but newer bioinformatic approaches as described above (see section 1.5.2) may go some way towards a solution.

### 1.7 Therapeutic modulation of the microbiome in RA

Current understanding of the microbiome in RA does not feasibly allow development of therapeutics at this stage. Moving forward, there is potential in the future for the microbiome to be useful as a target in RA, either as a target for modulation, or as a biomarker of potential for disease progression in arthralgia. Modulation may be possible via pro-biotics comprised of bacteria with beneficial functions, or precision editing, for example through use of bacteriophages. Given that RA seems to associate more with abundance of pathogenic bacteria, as opposed to a deficit in beneficial bacteria, precision editing of the microbiome may be a more likely option, for example through use of bacteriophages.

Current understanding of the RA microbiome is at an early stage, and as would be expected the pre-emptive trials of general use, non-RA specific probiotics have been inconclusive [12]. Use of the microbiome as a biomarker, to detect those at greater risk of progression from arthralgia to RA, is also feasible – if microbiome changes are present before onset of disease, and would allow for earlier intervention in these patients to improve clinical outcome [3].

## 2 Conclusions

There are compelling associations between the microbiome and RA, although the current evidence is far from conclusive that the microbiome causes RA. Strategic future studies replicating previous findings and addressing the gaps in the current knowledge are required. In particular it will be important to determine the influence of disease modifying RA medication on the microbiome. Host genetics may provide the link between the microbiome and RA and is a particular challenge to address, although current findings are suggestive of an important influence which may be mediated by the host immune system which could be ameliorated. A better understanding of whether associations described thus far are confounded by host genetics will shed further light on the role of the microbiome in rheumatoid arthritis.

## Funding

Twins UK receives funding from the Wellcome Trust European Community’s Seventh Framework Programme (FP7/2007-2013 to TwinsUK); the National Institute for Health Research (NIHR) Clinical Research Facility at Guy's & St Thomas' NHS Foundation Trust and NIHR Biomedical Research Centre based at Guy's and St Thomas' NHS Foundation Trust and King's College London. Cristina Menni is funded by the MRC AimHy (grant MR/M016560/1), Frances M.K Williams and Philippa M. Wells are funded by Arthritis Research UK Special Strategic Award (grant 21227), Claire J. Steves is funded by the Wellcome Trust (grant WT081878MA).
